# Effects of 60 minutes of hyperoxia followed by normoxia before coronary artery bypass grafting on the inflammatory response profile and myocardial injury

**DOI:** 10.1186/1477-5751-11-14

**Published:** 2012-09-14

**Authors:** Inga Karu, Peeter Tähepõld, Arno Ruusalepp, Kersti Zilmer, Mihkel Zilmer, Joel Starkopf

**Affiliations:** 1 North Estonia Medical Centre, Clinic of Anaesthesiology, Tallinn, Estonia; 2 Department of Anaesthesiology and Intensive Care, University of Tartu, Puusepa 8, Tartu, Estonia; 3 Department of Cardiology, University of Tartu, Tartu, Estonia; 4 Tartu University Hospital, Tartu, Estonia; 5 Institute of Biochemistry, Centre of Excellence for Translational Medicine, University of Tartu, Tartu, Estonia

**Keywords:** Coronary artery bypass grafting, Preconditioning, Hyperoxia, Troponin T, Cytokine, Interleukin, Tumor necrosis factor alfa, Interferon gamma

## Abstract

**Background:**

Ischemic preconditioning induces tolerance against ischemia-reperfusion injury prior a sustained ischemic insult. In experimental studies, exposure to hyperoxia for a limited time before ischemia induces a low-grade systemic oxidative stress and evokes an (ischemic) preconditioning-like effect of the myocardium. We hypothesised that pre-treatment by hyperoxia favours enchanced myocardial protection described by decreased release of cTn T in the 1^st^ postoperative morning and reduces the release of inflammatory cytokines.

**Methods:**

Forty patients with stable coronary artery disease underwent coronary artery bypass grafting with cardiopulmonary bypass. They were ventilated with 40 or >96% oxygen for 60 minutes followed by by 33 (18–59) min normoxia before cardioplegia.

**Results:**

In the 1^st^ postoperative morning concentrations of cTnT did not differ between groups ((0.44 (0.26-0.55) ng/mL in control and 0.45 (0.37-0.71) ng/mL in hyperoxia group). Sixty minutes after declamping the aorta, ratios of IL-10/IL-6 (0.73 in controls and 1.47 in hyperoxia, p = 0.03) and IL-10/TNF-α (2.91 and 8.81, resp., p = 0.015) were significantly drifted towards anti-inflammatory, whereas interleukins 6, 8and TNF-α and interferon-γ showed marked postoperative rise, but no intergroup differences were found.

**Conclusions:**

Pre-treatment by 60 minutes of hyperoxia did not reduce postoperative leak of cTn T in patients undergoing coronary artery bypass surgery. In the hyperoxia group higher release of anti-inflammatory IL-10 caused drifting of IL-10/IL-6 and IL-10/TNF-α towards anti-inflammatory.

## Background

Over 1 million coronary artery bypass grafting (CABG) operations are performed annually. In a large majority of patients the postoperative course is uneventful, *i.e*. without symptoms of major myocardial injury or systemic organ dysfunction. But in a small fraction of patients cardiac surgery results in a perioperative myocardial infarction, which is the major complication associated with morbidity and mortality, or excess systemic inflammatory response manifesting clinically as acute organ dysfunction. The concept of (ischemic) preconditioning-like cardioprotection induced by pre-treatment with hyperoxia has been reviewed recently [1]. Shortly, minimal concentration of oxygen in the inspired gas mixture evoking protective effect before subsequent ischemia in the rat heart is 80% and the most efficient > 95%, with the duration of 30 minutes for mice and 60 minutes for rats [2-7]. Hyperoxia improves recovery of postischemic contractile function, reduces infarct size both in normal and atherosclerotic hearts of experimental animals, reduces incidence of ischemia-reperfusion induced arrhythmias and apoptotic cell death [2,4,6,8-10]. This preconditioning-like effect is at least partly explained by a low-grade oxidative stress due to hyperoxic exposure, which activates various protective mechanisms (antioxidant enzymes, etc.) in the myocardium. As a consequence ‘readiness’ to cope with the oxidative stress during reperfusion is achieved.

In the first clinical study investigating the cardioprotective potential of hyperoxia during cardiac surgery, we employed hyperoxic exposure for about 130 minutes immediately before cardiopulmonary bypass (CPB), but could not detect protective effect [11]. The absence of time period between hyperoxic exposure and cardioplegia, during which the protective mechanisms could be activated, could have been one possible explanation for this negative observation. On that background, we designed the protocol for the present study, which includes a normoxic window between hyperoxic exposure and beginning of the CPB.

Cardiac surgery results in intense inflammatory response caused predominantly by surgical trauma, CPB and reperfusion injury. The effect of different cytokines in the heart is depending on different factors such as time of exposure and concentration [12,13]. Greater serum concentrations of tumour necrosis factor (TNF)-α and lower serum levels of interleukin (IL)-10 have been associated with increased prevalence of complications after CPB and may be indicative of a prominent proinflammatory state [14].

On that background, we aimed to evaluate cardioprotection conferred by adminisration of > 95% oxygen for 1 hour followed by normoxia up to initiation of CPB. Based on the previous clinical study and animal experiments we hypothesised that such pre-treatment provides enhanced myocardial protection described by decreased release of cardiac troponin T (cTn T) in the 1^st^ postoperative morning (primary endpoint of the study) and reduces the release of inflammatory cytokines (secondary endpoints of the study). For complex evaluation of dynamics of the inflammatory response, a spectrum of pro- and anti-inflammatory cytokines was assessed.

## Methods

### Patients

The investigation conforms to the principles outlined in the Declaration of Helsinki. Ethical approval for this study was provided by the Ethics Review Committee on Human Research of the University of Tartu and a written informed consent was obtained from all patients. Forty adult patients scheduled for isolated primary elective CABG with at least 3 distal anastomoses were included and randomised into the control (n = 20) and hyperoxia (n = 20) groups. The exclusion criteria were as follows: (1) preoperative infusion of vasoactive or inotropic medications, (2) diabetes mellitus treated either with insulin or oral medications, (3) hepatic, renal (serum creatinine > 150μmol/L) or pulmonary disease.

All medications, except salicylates were allowed until the day of surgery.

### Study protocol

After induction of anesthesia and intubation of the trachea, patients were randomly allocated to receive either 40% or > 96% oxygen for 60 minutes (ventilator: Siemens KIONi, Siemens-Elema AB, Sweden). Thereafter the mixture of oxygen and air was adjusted until the beginning of CPB to obtain arterial pO_2_ levels in the range of 100–150 mmHg. The fraction of oxygen in inspired gas mixture was continuously monitored by the gas analyser of the patient monitor Siemens SC9000XL (Dräger Medical System, Inc., Danvers, USA).

Arterial blood gases were analysed (Radiometer ABL 700 series, Radiometer Medical A/S, Copenhagen, Denmark) 10 and 60 minutes after randomisation and 10 and 60 minutes after the discontinuation of CPB.

### Anesthesia and operative procedure

Standardised intravenous anesthesia (midazolam, fentanyl, propofol, pancuronium) was used in all cases. Volatile anaesthetics were not used in order to avoid the confounding effect of a preconditioning-like state evoked by these agents. Nitroglycerine infusion (at least 1.0 mg/h) was started after the induction of anesthesia. CPB was performed with a roller pump (Maquet Critica Care AB, Solna, Sweden) and a membrane oxygenator (Maquet Quatrox-I, Hirrlingen, Germany) under mild hypothermia (nasopharyngeal temperature 35–36 °C). Warm blood cardioplegia was given antegradely into the aortic root. Infusion was repeated at least once every 15–20 minutes. Distal anastomoses were performed under a single aortic cross-clamp while proximal anastomoses were performed under single side-clamp. After CPB the pulmonary capillary wedge pressure was kept above 8 mmHg with the infusion of crystalloid and/or colloid solutions.

### Hemodynamic measurements

Thermodilution pulmonary artery catheter was inserted after induction of anesthesia. Heart rate, mean arterial, central venous, pulmonary artery and pulmonary capillary wedge pressure and cardiac output were recorded at baseline (before sternotomy) and 15 minutes, 1, 2, 4, 6, 9, 12 and 18 hours after discontinuation of CPB. Cardiac index (CI), right and left ventricular stroke work indices and systemic and pulmonary vascular resistance indices were calculated using standard formulas.

### Biochemical markers

Blood was sampled from the radial artery cannula before induction of anesthesia and 1, 6, 18 and 40 hours after declamping the aorta. Blood was centrifuged immediately after sampling and serum stored at −80 °C until analyses.

cTn T was measured using STAT (Roche) electrochemiluminescence immunoassay on „ECLIA“; Analyzer: Cobas e 411.

Interleukin-1α, IL-2, IL-4, IL-6, IL-10, TNF-α, and interferon gamma (IFN-γ) were analyzed with the Cytokine and Growth Factors High-sensitivity Array of the automated biochip immunoassay system, Evidence Investigator™ (Randox Laboratories Ltd., UK).

### Statistical analysis

We chose a difference between means of cTn T values in the 1^st^ postoperative morning of 0.4 ng/mL as clinically important. For a significance level (alpha) of 0.05 (two-tailed) and 80% power we calculated that 20 patients in each group were needed.

Patient data were analysed using Student’s *t*-test or Fisher’s exact test, as appropriate.

Hemodynamic and normally distributed biochemical data were evaluated using analysis of variance (ANOVA) for repeated measures and represented as the mean (standard deviation). Part of the biochemical data (incl. the ratios) showed non-normal distribution and were analysed using non-parametric repeated measures (Friedman) ANOVA and the Mann–Whitney *U*-test; the results are represented as the median (interquartile range). In case of significant Friedman test results, Wilcoxon Matched Pairs test was used for post hoc analysis. All tests were two-sided and p < 0.05 was considered to be significant.

## Results

### Demographic data

Patients’ characteristics are given in Table 1. There were no inter-group differences regarding age of the patients, ischemic time and number of grafted vessels. Significantly higher number of patients in the control group received calcium channel blocking drugs, otherwise the preoperative usage of medications did not differ between groups. Inotropic support (dobutamine as the agent of first choice) was administered in case of cardiac index <2.5 L min/m^2^, systolic blood pressure <90 mmHg and pulmonary capillary wedge pressure >8 mmHg.

**Table 1:** Characteristics of patients, oxygenation and surgical data

**Variable**	**Controls**	**Hyperoxia pretreated**	**p-value**
	**(n = 19)**	**(n = 20)**	
Age (years)	65 (8)	66 (11)	0.89
Gender (male/female)	15/5	14/5	0.67
Preoperative medications			
Ca-channel blockers, n (%)	6 (31.5)	1 (5)	0.01
Nitrates, n (%)	12 (63)	8 (40)	0.13
β-blockers, n (%)	17 (89)	14 (70)	0.13
ACE-inhibitors, n (%)	14 (74)	10 (50)	0.11
Statins, n (%)	15 (79)	11 (55)	0.27
Preoperative ejection fraction (%)	58 (11)	53 (11)	0.16
p_a_O_2_ 10 min after intubation (mmHg)	130 (49)	402 (68)	<0.001
p_a_O_2_ 60 min after intubation (mmHg)	108 (27)	369 (75)	<0.001
Cross-clamping time (min)	47 (33)	46 (30)	0.89
Duration of cardiopulmonary bypass (min)	68 (36)	63 (27)	0.66
Number of vessels bypassed	3.3 (0.8)	3.5 (0.8)	0.50
Need for inotropic support, n (%)	7 (37)	6 (30)	0.80

Values are provided as incidence (%) or mean (SD).

One patient was excluded due to missing hemodynamic measurements and blood sample at one time point, so results of 19 patients in the control group were analysed.

All patients experienced an uneventful recovery; *i.e*. there was no mortality or serious morbidity in this patient population.

### Myocardial necrosis

**Figure 1:**
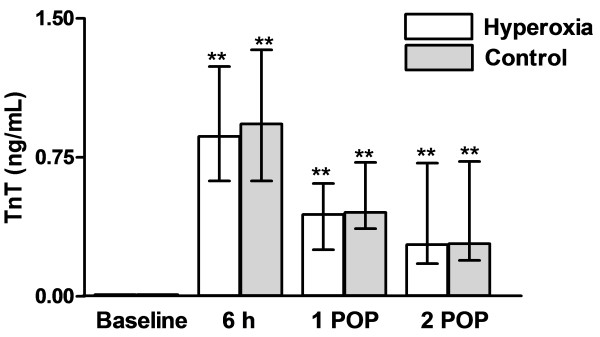
**Concentrations of cTn T at baseline, 6 h after declamping the aorta and in the 1**^**st**^**and 2**^**nd**^**postoperative mornings (POP).** Data are given as median with interquartile range. ** p < 0.01 in comparison with baseline.

Myocardial necrosis was not detectable preoperatively. In the 1^st^ postoperative morning the concentrations of cTn T did not differ between groups (0.44 (0.26-0.55) ng/mL in the control and 0.45 (0.37-0.71) ng/mL in the hyperoxia group) (Figure1). No significant differences were found between groups.

### Inflammation

#### Proinflammatory markers

Values of *interleukin-1α* (0.16 (0.15) pg/mL in the control and 0.13 (0.08) pg/mL in the hyperoxia group) and *interleukin-1β* (0.83 (0.76) pg/mL, and 0.94 (0.71) pg/mL, respectively) did neither differ preoperatively between groups nor change significantly during the study period. The values of *interleukin-2* did not differ between groups ((1.87 (0.57) pg/mL in controls *vs* 1.90 (1.02) pg/mL in hyperoxia pre-treated patients) perioperatively (data not shown).

**Figure 2:**
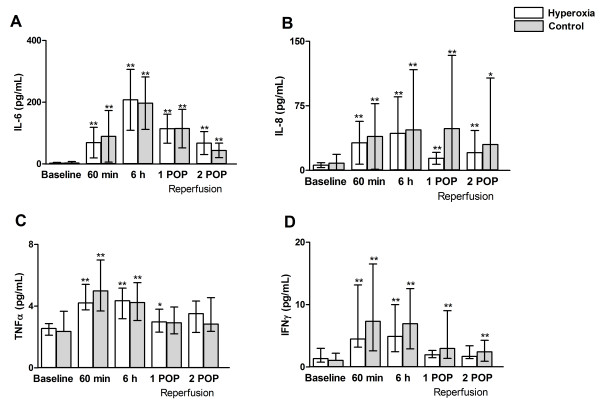
**Concentrations of proinflammatory cytokines (IL-6 – panel A, IL-8 – panel B, TNFα – panel B, IFNγ – panel D) at baseline, 6 h after declamping the aorta and in the 1**^**st**^**and 2**^**nd**^**postoperative mornings (POP).** Data are given as mean (SD) – panels **A**, **B** and as median (interquartile range) – panels **C**, **D**. ** p < 0.01 in comparison with baseline, * p < 0.05 in comparison with baseline.

*Concentrations of IL-6,8, TNF-*α and IFN-γ increased after cardiopulmonary bypass, but this was not affected by pre-treatment by hyperoxia (Figure2).

#### Anti-inflammatory markers

**Figure 3:**
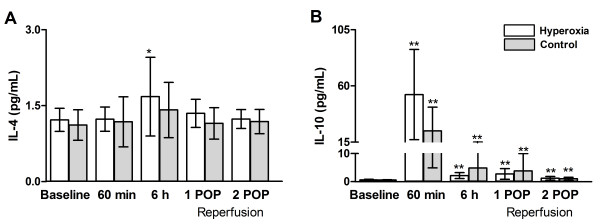
**Concentrations of anti-inflammatory cytokines (IL-4 – panel A, IL-10 – panel B) at baseline, 6 h after declamping the aorta and in the 1**^**st**^**and 2**^**nd**^**postoperative mornings (POP).** Data are given as mean (SD). ** p < 0.01 in comparison with baseline, * p < 0.05 in comparison with baseline.

*Iinterleukin-4* did not show any systemic changes over the time (Figure3, panel A).

*Interleukin-10,* in contrast, peaked 60 minutes after declamping the aorta and declined already by the 6^th^ post-declamping hour in both groups. Still, when compared to baseline, the values remained significantly elevated for 2 postoperative days (Figure3, panel B).

#### Ratios of pro- and anti-inflammatory markers

**Figure 4:**
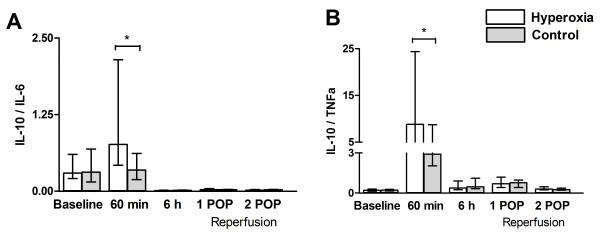
**Ratio of IL-10/IL-6 (panel A) and IL-10/TNFα (panel B) at baseline, 6 h after declamping the aorta and in the 1**^**st**^**and 2**^**nd**^**postoperative mornings (POP).** Data are given as median with interquartile range. * p < 0.05 in comparison with baseline.

Highest *IL-10 to IL-6 ratio* was detected at 60^th^ minute after declamping (0.73 in the controls and 1.47 in the hyperoxia group, p = 0.03). Thereafter the values in both groups were decreased to even below the baseline (Figure4, panel A). *IL-10 to TNF-α ratio* behaved in a similar manner, showing maximal values and significant difference between groups at 60^th^ minute after declamping (2.91 in the controls and 8.81 in the hyperoxia group, p = 0.015) and decreasing thereafter (Figure4, panel B). Higher IL-10 to IL-6 ratio in the hyperoxia group was mainly caused by higher IL-10 levels.

### Hemodynamic measurements

Preoperative mean CI was 2.2 (0.4) in the control and 2.1 (0.7) L min/m^2^ in the hyperoxia group. After CPB the CI, as well as left and right ventricular stroke work indices were not significantly different from with the baseline and no intergroup differences were found.

## Discussion

Present study demonstrates that pre-treatment with 60 minutes of hyperoxia followed by 33 (18–59) min normoxia before CPB does not afford protection against myocardial injury (cTn T release or myocardial function) associated with coronary artery bypass grafting. This is in accordance with our previous study where a different protocol of hyperoxic pretreatment was implemented [11]. We established that surgery was associated with a well-defined release of systemic inflammatory mediators, and in patients ventilated with >95% oxygen before CPB the ratios of IL-10/IL-6 and IL-10/TNF-α were drifted towards anti-inflammatory. It could be a reflection of changes in the inflammatory response network/profile due to pre-treatment with hyperoxia.

By now we have tested two different times of hyperoxic exposure in clinical studies – 60 minutes followed by normoxia in the present and about 130 minutes immediately before CPB in a previous study [11]. We did not find clinically relevant reduction of myocardial injury (release of cardiac troponin), although this manipulation resulted in some changes in the inflammatory profile. There can be several reasons for that. First, it could be speculated that in case of modern myocardial protection techniques only minimal myocardial injury is caused by cardioplegic cardiac arrest and there is no need for activation of intrinsic protective mechanisms. The situation is different in animal experiments where myocardial protective techniques are not used and injury is therefore well described. Although we included patients with at least 3-vessel CABG surgery, the aortic cross-clamping time of about 46 minutes could have been too short. Also, in power analysis we expected reduction of cTn T by 0.4 ng/mLin, which probably overestimates cardioprotective effect of whatever hypothetical intervention in these particular patients. To detect a difference of 0.1 ng/mL (the clinical relevance of which remains questionable), a study with several hundreds of patients needs to be conducted. Secondly, in difference from mice and rats, hyperoxic exposure of one or two hours in humans might be too weak stimulus to activate protective pathways. There is no direct evidence to support or oppose this idea. Some speculations might be drawn from the inter-species comparisons of myocardial injury in relation to duration of ischemia. For example, in rodent models of myocardial infarction, 30–40 minutes of either regional or global myocardial ischemia is sufficient to induce an infarct size of 50% of the area at risk. In the porcine heart, significantly longer duration (60–90 minutes) of myocardial ischemia is required to achieve equivalent levels of infarction. Human myocardial infarcts generally require 90 minutes to become established [15]. Whether these data can be extrapolated to the exposure time of hyperoxia that is needed to activate endogenous cardioprotective mechanisms, is not clear. In addition, it has been shown recently that in comparison with human broncho-alveolar fluid proteome, oxidative stress response was selectively enriched only in mice [16]. Regarding the response to pretreatment by hyperoxia, interspecies differences in evoking protective effect have been described even in the hearts of mice and rats^5^. Thirdly, there is a possibility that the phenomenon of hyperoxia induced myocardial protection does not exist in humans at all. *In vitro* studies, however, suggest that human myocardium can be preconditioned [17]. Several *in vivo* surrogate models of ischaemic preconditioning have showed protective effect - preinfarction angina, repeated balloon inflations, intermittent aortic cross-clamping. Similar to these, hyperoxia has been shown to be a preconditioning evoking stimulus both in rodents and in human *in vitro* studies [2-10].

One factor that could also have an impact on the results is the preoperative usage of statins and angiotensin converting enzyme (ACE) inhibitors. Statins have shown to evoke cardioprotective effects both in animal models of ischemia-reperfusion injury and in clinical studies (reviewed by Ludman et al. [18]), and have the ability to reduce IL-6 and IL-8 after coronary surgery [19]. ACE inhibitor treatment is associated with a reduction of IL-6 response to CABG [20] and confers added myocardial protection during surgical revascularisation [21].

Observed activation of inflammation in response to CPB is well in accordance with previous studies. TNF-α is a proinflammatory cytokine, which plays a central role in initiating and sustaining inflammation [22]. It enhances oxidative stress in adult cardiac myocytes both by increasing reactive oxygen species generation as well as by decreasing antioxidants. Overexpression of TNF-α leads to cell injury due to excessive oxidative stress [23]. IL-10 demonstrates potent anti-inflammatory properties through inhibiting the production of TNF-α and other proinflammatory cytokines [24]. It has revealed to possess antioxidant like properties in situations where oxidative stress is increased [25].

IL-10 has been shown to inhibit the production of reactive oxygen species in isolated macrophages [25] and has been suggested to modulate TNF-α mediated, oxidative-stress- induced acute lung injury [14,26,27]. After elective cardiac surgery the ratio of IL-10/TNF-α messenger RNA has shown to be an independent predictor of outcome, and thus IL-10 may have a protective role after cardiac surgery [28]. Hence, an appropriate balance between IL-10 and TNF-α may be of crucial importance. In the present study we observed a temporary increase of IL-10/IL-6 and IL-10/TNF-α ratios in the hyperoxia pre-treated patients, which suggests a change of the inflammatory profile towards anti-inflammatory. This can be a circumambage that hyperoxia may reduce inflammatory response immediately after CPB. Changes in immune system occur in 2 phases after cardiac surgery [29]. The 1^st^ phase represents the pro- and anti-inflammatory reaction returning to normal by the 3^rd^ postoperative day. This is the cause of systemic inflammatory response with well known clinical manifestations as acute organ dysfunctions and failures. The 2^nd^ phase emerges on postoperative day 5 and is characterised mainly by anti-inflammatory type of reaction. The synthesis of IFN-γ is significantly reduced after cardiac surgery and postoperative immunosuppression phenomenon in cardiac surgical patients has been well described [30].

## Conclusion

Pre-treatment by hyperoxia for 60 minutes followed by normoxia did not reduce postoperative leak of cTn T in patients undergoing coronary artery bypass surgery, but drifted ratios of IL-10/IL-6 and IL-10/TNFα towards anti-inflammatory.

## Competing interests

The authors declare that they have no competing interests.

## Authors' contributions

JS was involved in designing the study and revised the manuscript critically. PT and AR collected data and helped with revision of the manuscript. IK collected and analysed data and wrote the paper. KZ was responsible for the biochemical analysis. MZ helped to interpret the data and revised the manuscript critically. All authors read and approved the final manuscript.
